# Prevalence of postpartum depression in Nuuk, Greenland – a cross-sectional study using Edinburgh Postnatal Depression Scale

**DOI:** 10.3402/ijch.v72i0.21114

**Published:** 2013-08-05

**Authors:** Iben Motzfeldt, Sabina Andreasen, Amalia Lynge Pedersen, Michael Lynge Pedersen

**Affiliations:** 1 Queen Ingrid Centre for Primary Health Care, Nuuk, Greenland; 2 Clinic of Psychology, Nuuk, Greenland; 3 Greenland Centre for Health Research, University of Greenland, Nuuk, Greenland

**Keywords:** postpartum depression, Inuit, Edinburgh Postnatal Depression Scale

## Abstract

**Objective:**

The aim of this study is to estimate the prevalence of postnatal depression in Nuuk, Greenland.

**Study design:**

Cross-sectional study.

**Methods:**

The primary health care system in Nuuk initiated a project aiming to screen new mothers for depression using the Edinburgh Postnatal Depression Scale (EPDS). EPDS has a range on a scale from 0 to 30. All mothers residing in Nuuk who had given birth in 2011 were included in the study group. The screening was performed by health care visitors approximately 3 months following birth. Mothers who scored 13 points or above were defined as having possible postpartum depression (PPD). These mothers were then referred to a physician. A score at or less than 8 was defined as normal, whereas an intermediate score from 9 to 12 indicated a need for an extra visit.

**Results:**

During 2011, a total of 217 mothers gave birth in Nuuk. Of them, 80.2% (174) were screened for PPD using EPDS. Fifteen mothers scored 13 points or above corresponding to a prevalence of possible PPD at 8.6% (15/174). Seventy-nine percentage scored less than 9 points (137/174), whereas 15% (22/174) scored from 9 to 12 points.

**Conclusion:**

PPD seems to be a common problem in Nuuk, Greenland. EPDS seems to be a valuable tool in identifying women with PPD and vulnerable mothers with extra needs for support in a Greenlandic context. Continual routine screening is recommended.

## Background

Postpartum depression (PPD) is one of the most common conditions among women in the puerperal period (1–4). The prevalence of PPD is currently considered to be from 10 to 15% (2). However, considerable variability has been reported worldwide with prevalence of PPD ranging from almost 0% to more than 70% (2,3). In countries such as Singapore, Malta, Malaysia, Austria and Denmark, there are very few reports of PPD or postpartum-related depressive symptoms, whereas in other countries (e.g. Brazil, Guyana, Costa Rica, Italy, Chile, South Africa, Taiwan, Korea) reports on postpartum depressive symptoms are very prevalent (2). Also among people living in the Artic, PPD has been reported. A recent study from Canada using The Edinburgh Postnatal Depression Scale (EPDS) reported a prevalence of PPD as high as 29%, with indigenous people having a higher prevalence than non-indigenous people (3–5). However, the prevalence of PPD in Greenland is unknown.

PPD is a painful condition in which the mothers may experience feelings of loneliness, anxiety, hopelessness and loss of control at a time when expectations of joyfulness are anticipated (3). Depressive symptoms in the postpartum period are often strongly influenced by concern about the child and the demands of motherhood (1). Furthermore, PPD has considerable negative impact on the children, husbands/partners and family (6). Several studies have demonstrated decreased prevalence of breastfeeding among mothers suffering from PPD compared to non-depressive mothers (7–9).

It is well documented that PPD poses a risk for the mother–infant relationship and infant cognitive and emotional developmental outcome (10–17). The negative effects are seen both in early infancy with reduced verbal and visual communication and later in childhood as insecure-avoidant attachment and reduced cognitive, emotional, verbal and social skills (15). Furthermore, increased risk of affective disorders in the offspring has been reported (16).

Treatment for depression has been found to be effective and generally safe during pregnancy and while breastfeeding (18). The risks and benefits of treatment must be carefully evaluated and balanced with the risk of no treatment (18).

However, PPD continues to be underdiagnosed and undertreated despite the mothers’ regular contact with the health care system during pregnancy and early motherhood (1,4,18).

In some health care systems in Australia and the United States, this deficiency is addressed by routine screening for PPD (1). EPDS (19,20) is the most widely used screening tool for PPD (2,21). Screening for PPD may also be relevant in Greenland, where the prevalence of PPD in Greenland is unknown.

The aim of this study is to estimate the prevalence of postnatal depression in Nuuk, Greenland.

## Materials and methods

The study is designed as a cross-sectional study of the whole population in Nuuk, the capital city, representing almost 30% of the population of Greenland (22).

### Setting

Greenland is the largest island in the world covering an area of more than 2 million square kilometres. The country is sparsely populated with approximately 56,000 inhabitants living widely spread along the coast (22). Approximately 90% of the population is of Greenlandic origin, whereas 10% are immigrants (mostly Danes) (23).

All deliveries in Nuuk are performed at Queen Ingrid's Hospital's Department for Gynaecology and Obstetrics. All mothers and infants in Nuuk are offered routine health care service including home visits by specially trained nurses from Queen Ingrid Health Care Centre in Nuuk.

### Study population

A project aiming to screen all mothers affiliated with the clinic was initiated on 1 January 2011. All mothers who spoke Greenlandic or Danish, were residents in Nuuk, and had given birth in Nuuk during 2011 were offered a screening for PPD including in a routine home visit approximately 3 months after birth. The screening was performed by health care nurses using EPDS (19,20).

### Edinburgh Postnatal Depression Scale

EPDS has been used and validated in many countries and cultures worldwide (2,20,24,25). We used the Danish version (26) for the women speaking primarily Danish. The Danish version was translated and back-translated from the original English version and has been used in a Danish study including more than 5,000 mothers (27). In our study, the Danish version of EPDS was translated into Greenlandic and back-translated to check whether the questions elucidated the intended information. EPDS consists of 10 items; the whole test is performed within 5 min. Each item is scored with 0–3 points. The total score ranges from 0 to 30 points.

### Procedures in the study

In this study, a score less than 9 points was considered normal. Mothers who scored 13 points or above were defined as having possible PPD (19,26,27). These mothers were then referred for further evaluation and intervention by the general practitioners at Queen Ingrid Health Care Centre. Mothers who scored from 9 to 12 had indication of minor signs of depression and were offered supporting follow-up visits by the health care nurses.

Basic information about maternal age, gestational age at delivery, marital status and first-time motherhood was collected through review of the perinatal medical record.

### Analysis and statistics

Chi-square tests were used to compare frequencies. Means were compared using t-test.

p-Values less than 0.05 indicated statistical significance. Estimates were calculated with 95% confidence intervals (CIs).

### Ethics

This study was approved by the local ethical committee for health research in Greenland. The mothers were recruited by specially trained nurses as part of routine home visits. Mothers were asked if they wanted to participate in a visit shortly after delivery and again in a visit approximately 2 months after birth. All mothers were informed orally and in written form that the study was voluntary and participants could be withdraw at any time. All mothers in the study provided written consent.

## Results

**Fig. 1:**
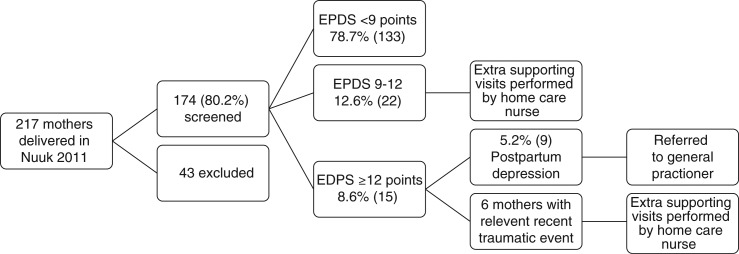
A flow chart illustrating the classification of mothers within the study.

A total of 217 mothers gave birth in Nuuk during 2011. Of these, 174 (80.2%) were screened using EPDS (see Fig. 1). Forty-three mothers were not screened for various reasons: 16 were either away for holiday or moved from the town, 4 were not home at the time for the visit, 2 lived outside Nuuk, 3 spoke neither Greenlandic nor Danish, 7 mothers put their children up for adoption, 1 mother had a stillborn child and 10 mothers refused participation. One-hundred and thirty-three mothers (78.7%) scored less than 9 points indicating no sign of PPD. Twenty-two mothers (12.6%) scored from 9 to 12 points. These mothers were followed with extra supporting visits by the home care nurses. None of these cases were referred to the general practitioner due to improvements during the follow-up period. Fifteen mothers scored 13 or more points indicating a possible PPD corresponding to a prevalence of PPD at 8.6% (95% CI: 4.5–12.8) (15/174). In 6 cases, the home care nurse identified a recent traumatic event in the mothers’ life; for example a recent, unexpected death among near relatives. These 6 mothers were not referred to the general practitioner, but were followed with offers of extra supporting visits by the home care nurses. In all cases, a new score performed 4–8 weeks after the first test measured normal with scores less than 9 points. Nine cases corresponding to a prevalence of PPD at 5.2% (95% CI: 1.9–8.5) were referred to the general practitioner for further treatment. In 8 cases, treatment for depression was addressed whereas 1 mother was followed with extra supporting visits by the home care nurses. Four mothers were treated by the general practitioner, 2 mothers were referred to the Department of Psychiatry at Queen Ingrid's Hospital and 2 mothers were advised to take up psychological therapy offered by the municipality.

Basic information for all mothers and for mothers scoring 13 or more points is shown in Table I. The mean age of the screened mothers was 28 years with no difference between mothers with a score at or above 13 and mothers with a score less than 13. First-time mothers were more likely to score less than 12 points than multiple-birth mothers (p=0.016). Unemployed and single mothers scored higher than employed and in couple mothers but the observed difference was not significant due to very small numbers in the sample.

**Table I:** Clinical and demographical variables of the mothers

Characteristic or variable	All mothers (N=174), mean (SD; range)	Mothers with EPDS at or above 13 (N=15), mean (SD; range)	Mothers with EPDS less than 13 (N=159), mean (SD; range)	p
Age (years)	28 (5.4; 17–42)	27 (4.6; 20–34)	29 (5.4; 17–42)	0.198
Gestational age (weeks)	38.8 (1.9; 27–42)	38.8 (2.0; 27–42)	38.5 (1.0; 37–40)	0.589
EPDS (points)	6.0 (4.3; 0–22)	5.1 (3.1; 0–12)	16.2 (2.8; 13–22)	<0.001
Percentage (number)				
First-time mothers	49.4 (86)	20.0 (3)	52.0 (83)	0.016
Unemployed	9.2 (16)	20.0 (3)	8.2 (13)	0.133
Single mothers	8.6 (15)	20.0 (3)	5.6 (9)	0.100

## Discussion

The prevalence of PPD in Nuuk based on EPDS scores was 8.6% (95% CI: 4.5–12.8) in 2011, indicating that PPD is also a common problem in Greenland and that EDPS can be used as a screening tool.

The strength of this study is that more than 80% of all the mothers who have given birth in Nuuk 2011 were included. The study has evaluated the use of EPDS implemented in a clinical setting rather than in a specially designed research project. This resulted in data applicable to the Greenlandic context.

The screening in this study was performed 3 months after birth. Since the peak time for PPD is 4–6 weeks after birth (4), the incidence documented in this study may be underestimated. Comparisons with other studies worldwide should be conducted with reservation. In addition to different screening times, different diagnostic cut-off values have been applied across studies. We used the cut-off value used in the Danish version of EPDS (26,27) and in the original EPDS (19). This is higher than that used in some studies which will lead to a lower prevalence compared to studies with a lower cut-off value.

Another limitation in the study is the lack of a proper validation of the Greenlandic version of EPDS. The EPDS has been validated in many cultural settings and languages, including Norwegian and Swedish (28,29), and no acceptable alternative is available at present.

The study sample is relatively small and the limited number of mothers involved influences the precision of the prevalence estimates. Thus, the statistical tests may lack sufficient power to identify differences between mothers with and without PPD according to EPDS. However, this does not influence the usefulness of EPDS as a screening tool. The use of EPDS seems to have helped the home care nurses in identifying both mothers with possible PPD as well as a group of vulnerable mothers with needs for extra temporary support. In addition to the 9 cases referred to the general practitioner for PPD, 22 mothers were offered extra visits by the home care nurse. This is one challenge from among competing priorities of home care nurses. However, the possibility of preventing the severe negative effects of PPD is crucial and may be very cost effective in a wider perspective. A systematic Cochrane meta-analysis suggests that psychosocial and psychological interventions are effective treatment options for women suffering from PPD (30).

Recent studies from Norway have demonstrated that public health nurses find EPDS easy to score and easy for mothers to complete (31). In addition, the nurses identified PPD more frequently than without EPDS (31). Supportive counselling based on non-directive counselling provided by health nurses was found to be an effective treatment method for PPD (32,33). Similarly, randomised studies from England indicate that specially trained health visitors using EPDS can identify PPD and deliver psychologically informed sessions with significant clinical effectiveness 6–12 months postnatally (34–36). Psychosocial and psychological interventions delivered during pregnancy can be effective in preventing postnatal depression (37).

In this study, none of the 22 mothers who were offered extra home visits developed depression within the observation period suggesting a positive effect of that intervention, although no control group existed. A recent systematic review has documented that PPD screening improves recognition of the disorder (4). The screening should be followed by extensive care and adequate treatment to improve the clinical outcome (4). Screening for PPD in a Greenlandic context with gradual intervention – including extra supporting visits in the intermediating scoring group and referrals to general practitioner or psychological intervention in the high scoring group with possible PPD – seems to be feasible and also evidently well-founded.

The prevalence of PPD demonstrated in this study is similar to the prevalence of 5.5% reported in Denmark 4 months after delivery using the same cut-off score (27), and in the prevalence reported in Norway where 10% was reported to have PPD 6 weeks after delivery (38). In the Norwegian study, a lower cut-off value at 10 points instead of 13 points was used to define PPD (38). The prevalence demonstrated in this study is a little lower than the global average at 10–15% (1–3).

In some ways, it is surprising that the prevalence of PPD was similar to the prevalence in Denmark and Norway. Mental vulnerability has been previously well documented among young Greenlanders and other indigenous people in the circumpolar area (39–41). As an example, youth suicide rates are very high in many parts of the Arctic, particularly in Greenland and Alaska (39). However, comparing the prevalence of PPD between countries must take into consideration several limitations (2,21). The variability in reported prevalence of PPD might be due to cross-cultural variables, reporting style, differences in perception of mental health and the stigma attached to mental illness, differences in socio-economic environments (e.g. poverty, levels of social support or its perception, nutrition, stress) and biological vulnerability factors (21). The influence of Danish and Nordic cultural, linguistic and health care traditions could provide part of that explanation.

## Conclusion

PPD is a common health care problem in Greenland that can be addressed by using EPDS as a screening tool in combination with a willingness to support the most vulnerable mothers and with offers of proper treatment to those with possible PPD. It is recommended to maintain the use of EPDS as a screening tool in Nuuk and to extend the use to the rest of Greenland. However, the screening should be performed earlier after birth to identify more mothers at the risk of developing PPD.
